# Over-Prescription and Overuse of Antimicrobials in the Eastern Mediterranean Region: The Urgent Need for Antimicrobial Stewardship Programs with Access, Watch, and Reserve Adoption

**DOI:** 10.3390/antibiotics11121773

**Published:** 2022-12-08

**Authors:** Maha Talaat, Sara Tolba, Enjy Abdou, Mohamed Sarhan, Mohamed Gomaa, Yvan J-F. Hutin

**Affiliations:** 1 Antimicrobial Resistance and Infection Prevention and Control Unit, Universal Health Coverage, Communicable Diseases Department, Eastern Mediterranean Office, World Health Organization, Cairo P.O. Box 7608, Egypt; 2 Department of Universal Health Coverage/Communicable Diseases Prevention and Control, Eastern Mediterranean Office, World Health Organization, Cairo P.O. Box 7608, Egypt

**Keywords:** point prevalence survey, antimicrobial use, Eastern Mediterranean region, AWaRe classification

## Abstract

Excessive antimicrobial use contributes to the development of antimicrobial resistance. In the Eastern Mediterranean region (EMR), there is dearth of information on the prevalence of antimicrobial use in patients hospitalized in acute healthcare settings, clinical indications, types of antimicrobials prescribed, and quality indicators for prescriptions. Between September and December 2019, seven countries in the EMR conducted a standardized point prevalence survey. All patients present in the hospital wards at 8 a.m. on the day of the survey constituted the sample population. We collected data, including patient characteristics, antimicrobials received, therapeutic indication according to predefined lists, and markers of prescribing quality. The survey included data from 139 hospitals in seven countries. Among the 19,611 inpatients surveyed, 11,168 patients received at least one antimicrobial {crude prevalence: 56.9% (95%CI: 56.2–57.6%). The top three classes of antimicrobials prescribed were third-generation cephalosporins (26.7%), beta-lactam penicillins (18.1%), and imidazole derivatives (n = 1655, 9.8%). Carbapenems were most frequently prescribed for the treatment of healthcare-associated infections. Compliance with quality indicators of antimicrobial use was limited where treatment guidelines were available for 41% of antimicrobial prescriptions and targeted antimicrobial treatment represented 21% of therapeutic indications. Overall hospital antimicrobial use was high in countries of the EMR, pointing to the need to design and implement context-specific antimicrobial stewardship programs to optimize antimicrobial use and reduce antimicrobial resistance.

## 1. Introduction

Antimicrobial resistance (AMR) poses a major global threat to human health around the world. In 2019, the Global Burden of Disease project of the Institute for Health Metrics and Evaluation (IHME) estimated that drug-resistant bacterial infections were associated with 4.95 million deaths, including 1,270,000 deaths attributable to AMR [1]. The overuse of antimicrobials in humans, animals, and plants drives the development and emergence of AMR [2]. Reducing antibiotic use and consumption prevents the emergence of resistance, particularly given the long timescale and number of resources necessary for the development of new antibiotics [3]. In 2015, the University of Antwerp launched a standardized global point prevalence survey method. Hospitals worldwide from high and low middle-income countries participate in web-based tools to measure antimicrobial prescribing practices. This results in the availability of data on the magnitude and indications of antimicrobial use globally, which are used to benchmark the prevalence of use across countries and hospitals [4]. Repeated surveys on antimicrobial use over time provide data on the impact of implementing antimicrobial stewardship (AMS) programs and interventions [5]. 

AMS programs are one of the key actions that identify targets for quality improvement and improve the clinical outcomes of patients. AMS is one of three pillars of an integrated approach to health system strengthening, including infection prevention and control and medicine and patient safety. In 2017, the WHO developed the AWaRe classification as a simple stewardship tool to monitor prescribing patterns of antibacterials of different spectra at local, national, and global levels. AWaRe classifies antibacterials into 3 categories: Access (narrow-spectrum), Watch (broad-spectrum), and Reserve (last resort), taking into account the impact of different antibiotic classes on antimicrobial resistance to emphasize the importance of their appropriate use. The WHO set a 60% national target of total antibiotic consumption in the Access category by 2023 [6,7]. 

Data on antimicrobial use are scarce in the EMR, particularly in LMIC; however, high levels of antimicrobial resistance are increasingly reported, where the median proportion of bloodstream infections (BSI) in 2019 was highest for carbapenem-resistant *Acinetobacter* spp. at 70.3% (IQR 62.4–81.3%), followed by *Klebsiella pneumonia* resistant to 3rd-generation cephalosporins (66.3%, IQR 54–3.8%) [8]. To understand how the imprudent use of antimicrobials contributes to antimicrobial resistance, we measured the prevalence of antimicrobial use in patients hospitalized in acute healthcare settings in select EMR countries, described clinical indications, the types of antimicrobials prescribed, and the quality indicators for antimicrobial prescription. 

## 2. Results

Seven countries (Tunisia, Pakistan, UAE, Jordan, Iraq, Sudan and Lebanon) participated in the point prevalence survey studies, including 139 acute care hospitals with a total number of 42,976 hospital beds. Public sector hospitals accounted for 50%, while 26% were private and 24% were from academia. 

### 2.1. Antimicrobial Prevalence by Hospital Size, Ownership, and Ward Specialty

**Figure 1:**
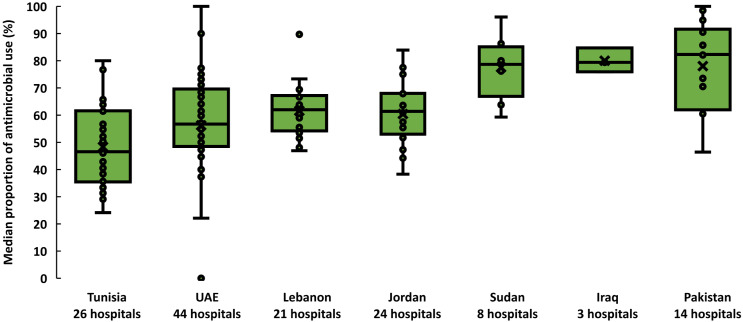
Prevalence of antimicrobial use across 139 hospitals, seven countries in the Eastern Mediterranean Region, 2019. Each dot represents the proportion of patients who received at least one antimicrobial in a hospital. Horizontal lines within boxes indicate medians, box tops and bottoms indicate interquartile ranges (middle 50% of data), and error bars (upper and lower whiskers) represent scores outside the middle 50%. UAE is the United Arab Emirates.

From September to December 2019, 19,611 inpatients were included. Of these, 11,168 patients received at least one antimicrobial {56.9% crude prevalence (95% CI: 56.2–57.6%)}. Across countries, the prevalence of antimicrobial use was heterogenous. The median proportion of patients prescribed antimicrobials ranged from 46.5% (interquartile range (IQR) 35.4–61.5%) in Tunisia to 82.3% (IQR 61.6–90.5%) in Pakistan (Figure 1). The 11,168 patients treated received 16,885 antimicrobials (1.5 antimicrobials per patient, range: 1.5–1.7). Out of the 11,168 patients who received at least one antimicrobial, 40.8% were admitted to medical wards, 28.6% were admitted to surgical wards, and 17.2% were admitted to pediatric and neonatology wards. Patients admitted to private hospitals were more likely to receive one or more antimicrobials (60.7%; 95% CI: 58.6–62.8%) than those admitted to public hospitals (57.5%; 95% CI: 56.5–58.6%) and academia hospitals (55.4%; 95% CI: 54.4–56.5%). In four countries, patients admitted to hospitals with fewer than 100 beds were more likely to receive antimicrobials (66%, 95% CI: 63–69) than others. 

### 2.2. Indications of Antimicrobial Prescriptions

**Figure 2:**
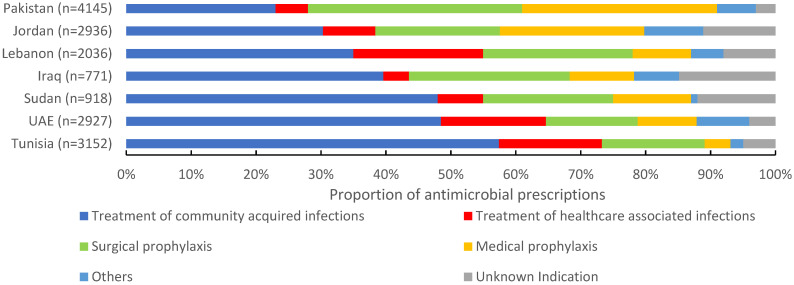
Indications of prescribed antimicrobials in 139 hospitals in seven countries in the Eastern Mediterranean Region, 2019 (n = 16,885). n = number of prescriptions.

**Figure 3:**
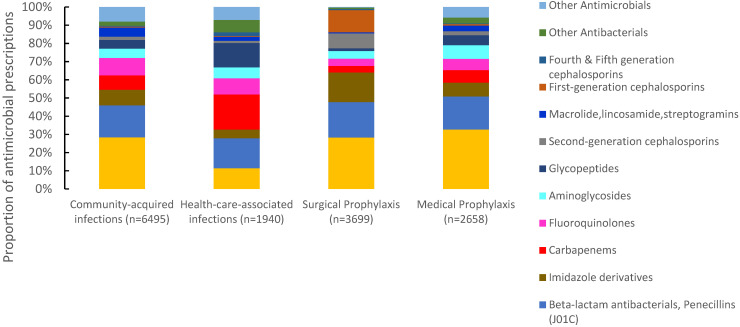
Distribution of prescribed antimicrobials according to clinical indications, seven countries in Eastern Mediterranean Region *, 2019. * Seven countries are Tunisia, United Arab Emirates, Lebanon, Jordan, Pakistan, Sudan, and Iraq. $ Other antimicrobials include systemic antivirals, antiprotozoals, intestinal anti-infectives, antimycotics. for systemic use and antimycobacterial. (n = number of prescriptions).

Of 16,885 antimicrobials prescribed, 38.5% (6495) were prescribed for community-acquired infections, 21.8% (3699) for surgical prophylaxis, 15.7% (2658) for medical prophylaxis, and 11.4% (1940) for the treatment of healthcare-associated infections (HAIs). The proportion of antimicrobials prescribed for the treatment of CAIs ranged between 23% in Pakistan to 57.9% in Tunisia. For surgical prophylaxis, Pakistan had the highest proportion of prescribed antimicrobials (32.9%), followed by Iraq (24.8%) and Lebanon (23.2%). Pakistan also had the highest proportion of prescribed antimicrobials (30.1%) for medical prophylaxis, and Tunisia the lowest (4%). For HAIs, Lebanon reported the highest proportion of prescribed antimicrobials (19.9%) and Iraq the lowest (4.4%) (Figure 2). Third-generation cephalosporins were the most prescribed group for CAIs (28.4%, 1844/6495), surgical prophylaxis (28.3%, 1046/3699), and MP (32.6%, 868/2658), with a wide variation among countries. Carbapenems were also prescribed for SP at 3.6% (134/3699) of the total prescribed antimicrobials. For HAIs, carbapenems were the most prescribed (19.3%, (375/1940), ranging from 11.8% in Iraq to 24.4% in Lebanon. First-generation cephalosporins accounted only for 12.2% (n = 515) of antimicrobials prescribed for surgical prophylaxis ranging from 0% in Iraq to 39.3% in UAE (Figure 3). 

### 2.3. Types of Prescribed Antimicrobial Groups

Of 16,885 antimicrobial prescriptions, antibacterials accounted for 95.2% (16,071) while antimycobacterial, antimycotics for systemic use, antiprotozoals and systemic antivirals represented only 4.8%. Table 1 presents the ten most common antimicrobial groups prescribed, with a total number of 15,391 prescriptions. The top four prescribed antimicrobial groups were third-generation cephalosporins (n = 4516, 26.7%), beta-lactam penicillins (n = 3056, 18.1%), imidazole derivatives (n = 1655, 9.8%) and carbapenems (n = 1385, 8.2%). Third-generation cephalosporins were the most prescribed antimicrobials in Iraq (38.4%), Pakistan (38.5%), Sudan (31.7%), and Jordan (28.6%). First- and second-generation cephalosporins accounted only for 3.1% (525) and 3.6% (602) of the total prescribed antimicrobials, respectively, with variation among countries (Table 1). 

### 2.4. AWaRe Classification

**Figure 4:**
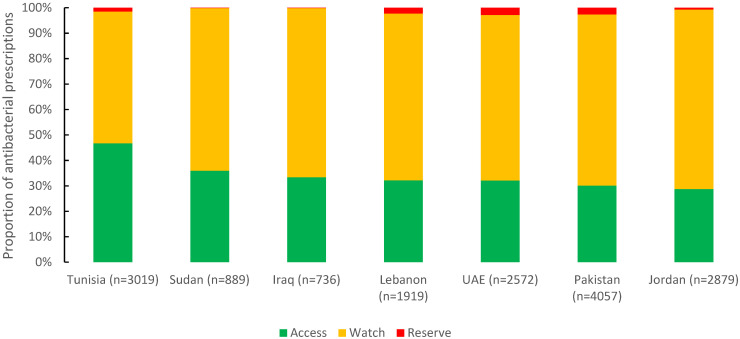
Distribution of prescribed antibacterial agents by AWaRe classification in seven countries in the Eastern Mediterranean Region, 2019 (n = 16,071).

Of 16,071 antibacterials prescribed, 34.1% were from the Access group (5470), 64.1% from the Watch group (10,308), and 1.8% from the Reserve group (293). Tunisia had the highest reported proportion of the Access group (46.7%), followed by Sudan (36%) and Iraq (33.4%). For the Watch group, Jordan (70.5%), Pakistan (67.2%), and Iraq (66.4%) were almost the same (Figure 4).

### 2.5. Antimicrobial Quality Indicators

Local treatment guidelines were available for 41% (6841) of antimicrobial prescriptions ranging from 7% in Iraq to 82% in UAE, while the overall compliance with guidelines was 74%. The reason documented for antimicrobial use was reported in 61% of prescription, and was highest in UAE (87%), followed by Lebanon (74%) and Tunisia (62%), and was lowest in Iraq (12%). The stop or review date of antimicrobial treatment was only available for 30% of documented antimicrobial prescriptions ranging from 3% in Pakistan to 72% in UAE. Out of 16,763 antimicrobial doses prescribed, 14,480 (86%) were injections, ranging from 74% in UAE to 92% in Sudan and Pakistan. Out of 9179 prescriptions indicated for therapeutic purposes, targeted antimicrobial treatment based on culture results represented only 21% with the highest in UAE (32%) and the lowest in Pakistan (6%) (Table 2).

## 3. Discussion

Overuse and misuse of antimicrobials facilitates the emergence of multidrug resistant organisms. The literature shows that antimicrobial stewardship programs are effective in optimizing antimicrobial use and therefore key to preventing the emergence of resistance while IPC programs prevent spread [2,3]. This study documented a high antimicrobial use of 56.9% (range from 39% to 78%) with variation of estimates across seven countries in the EMR. This high use is reflected by the high burden of AMR in many EMR countries, with an increasing trend over time [8]. The proportion of antimicrobial use at 56% is higher than the prevalence reported from 29 European countries (30.3%) [95% CI 29–31.6%] and similar to the prevalence reported from four Latin American countries (49.5%) [9,10]. The prevalence of antimicrobial use varied across countries. It was lowest in middle- and high-income countries, such as Tunisia, UAE, Lebanon, and Jordan (39% to 60%). These countries had prevalence comparable to high-income countries, including Switzerland (33%), Canada (34%), and Singapore (51%) [5,11,12]. In countries with limited healthcare system capacities, such as Iraq, Sudan, and Pakistan, the prevalence of antimicrobial use exceeded 70%. This high prevalence was similar to countries in Africa, e.g., Nigeria (80.1%), Botswana (70.6%), and Uganda (73.7%) [13,14,15].

Multiple factors drive the use of antimicrobials in a country. These depend on the disease burden estimates within a country and the cultural context related to increased patient demands for antibiotics. Physicians tend to overprescribe antimicrobials in countries with poor regulations on antimicrobial use and weak antimicrobial stewardship programs [12]. The implementation of antimicrobial stewardship programs is challenging in LMIC. There is limited training of physicians on AMS and no national treatment guidelines for infectious diseases that could be used as a reference to treating physicians. In countries with limitations on the use of the microbiology laboratory, there is always a tendency of physicians to provide empiric treatment and the use of broad-spectrum antibiotics to cover wide range of bacterial species [16]. The problem is more complicated in countries where national- and facility-level IPC programs are still evolving, which is the case in Sudan, Pakistan, and Iraq. In these countries, physicians tend to overprescribe antibiotics as surrogates for IPC to protect their patients from acquiring hospital infections [8,17]. 

Despite differences in the prevalence of antimicrobial use among countries, our analysis identified some patterns. CAIs were the most common reason for hospital antimicrobial use in most countries studied, as well as several countries in Africa and Europe (49.5%), accounting for approximately 40% of all antimicrobial treatment [9,18]. Antimicrobial use for HAIs in Tunisia, UAE, and Lebanon (16–20%) was comparable to Saudi Arabia (16.4%), Germany (20%), and Belgium (25.3%), but considerably higher than in other settings, e.g., ECDC survey (6%) and the GLOBAL-PPS survey (8.4%) [4,9,19,20,21]. The high antimicrobial use for medical prophylaxis in Jordan (22%) and Pakistan (30%), as well as the high antimicrobial use for unknown indications in Iraq (15%) and Sudan (12%), reflect the unnecessary prescriptions to patients without clear medical indication. The use of MP was less than 15% in Tunisia, UAE, Lebanon, Iraq, and Sudan, in line with the data from Belgium (5.9%), Switzerland (8.8%), and Canada (14.2%) [5,11,21].

The high use of third-generation cephalosporins in Jordan, Iraq, Sudan, and Pakistan (ranging from 25–38.4%) is a source of concern. They were commonly prescribed for surgical and medical prophylaxis, against recommendations from the WHO [22]. The high use of third-generation cephalosporins is comparable to reports from Indonesia (26.8%), Egypt (28%), and Russia (41.8%) [23,24,25]. This remarkable use has led to the increase in third-generation cephalosporin resistance in *Escherichia coli* and *Klebsiella pneumoniae*, causing bloodstream infections from 2017 (58%) to 2019 (65.4%) [8]. Several factors could explain this finding. First, treating physicians acknowledged the circulation of highly resistant pathogens that necessitate the use of broad-spectrum antibacterial agents to protect the patients. Second, knowledge of rational antibacterial prescription is limited. Third, narrow-spectrum antibacterials may be non-accessible [24,26,27]. Some countries report low use of third-generation cephalosporins, including Singapore (7.7%) and South Africa (10.7) [12,28].

In our report, the overall use of the antibacterials from the Access group fell short of the 60% target set by the WHO, ranging from 28.8% in Jordan to 46.7% in Tunisia [29]. Several high-income countries in Europe and the US reported a median use of 68% from the Access group through enhancing the use of narrow-spectrum antibiotics (France, Germany, Italy, Japan, Spain, Switzerland, United Kingdom, Northern Ireland, and United States of America) [30]. The use of the Watch group indicates a preference towards using broader spectrum antibacterials. The consequences of high use of the Watch group are reflected in the increasing burden of pathogens resistant to carbapenems such as *Acinetobacter* spp. and *Klebsiella pneumoniae* in several countries in EMR [8]. The use of Reserve antibacterials (e.g., colistin) was highest in UAE, Pakistan, and Lebanon (2.3–2.9%), exceeding the reported overall use in high-income countries (1.5%) [30]. Colistin is the last-resort treatment against multi-drug resistant Gram-negative pathogens and its high use contributes to the global emergence of colistin-resistant pathogens [31]. The limited availability and high price of the Reserve antibacterials may explain the low use of these medicines in Iraq (0.1%) and Sudan (0.1%) [32,33].

Our quality indicators could be used to set country-specific benchmarks for quality improvements and opportunities for AMS programs. First, the existence of national treatment guidelines was limited in most countries. Lack of clear guidance in clinical settings may explain the high antimicrobial prescription of treating physicians. However, compliance with hospital treatment guidelines—if available—was relatively high at 74%, which is higher than the compliance reported from Latin America, west and central Asia, and Africa [4]. Second, the documentation of the reason for prescription was high in UAE and Lebanon, reaching levels similar to Europe (81%), Asia (73%), and North America (85%) [4]. In other countries, documentation was less common. Low documentation poses a challenge for the effective review of the appropriateness of the antimicrobial prescriptions and impedes the follow up of stewardship activities. Third, documentation of the stop or review order of prescribed antimicrobials was limited across the countries (about a third), and may indicate the unnecessary prolonged use of antimicrobials, as well as absence of stewardship interventions e.g., de-escalation, shortening antibiotic treatment duration, and switching from IV to oral medication [34]. Fourth, parenteral administration was common (86%), similar to Asia, Latin America, and Europe (more than 80%). Third-generation cephalosporins account for a substantial proportion of broad-spectrum antibiotics, and some of them are available in oral format [4]. Replacing the intravenous therapy with oral medications might reduce rates of catheter-related complications, healthcare costs, and the duration of hospital stays. Fifth, targeted treatment represented a small proportion of the antimicrobial use for treatment purposes (21%), lower than in Belgium (37%) and Canada (40.4%) [5,21]. Limited microbiological capacities of the hospital laboratories, with a prolonged turnaround time of culture results and delayed communication of preliminary culture results to treating physicians may explain the low proportion of targeted treatments [35]. Overall, the challenges identified in this survey reflect the limitations in design, development, and implementation of AMS programs across contextually different economies. These AMS programs are still evolving, with major barriers; national leadership and engagement are lacking, human and financial resources and expertise are limited, infectious diseases are not a recognized specialty in many countries, health information systems do not monitor antimicrobial use, and microbiology capacity is basic [8,36]. 

Our study suffers from a number of limitations. First, we measured the prevalence of antimicrobial use using unique point estimates, which limits inferences. Second, the results were not corrected for difference in healthcare facility factors, patient case mix, variations in resistance levels, or institutional factors, all of which can influence antimicrobial use patterns. Third, not all countries conducted a nationally representative sampling design, which limited our capacity to make comparisons across countries. 

## 4. Materials and Methods

Between October and December 2019, we conducted cross-sectional point prevalence surveys in seven countries in the region using a standardized WHO Eastern Mediterranean protocol designed from the WHO protocol [37]. Countries participated on a voluntary basis, based on the interest and the capacity to implement the survey, generate data, and use evidence to guide antimicrobial stewardship program design and implementation. 

### 4.1. Study Design

Four countries—Tunisia, Lebanon, Jordan, United Arab Emirates (UAE)—selected a nationally representative sample of acute care hospitals. Three countries used a convenient sample (Sudan, Pakistan, Iraq). Each country completed the survey within 3 weeks among all participating hospitals and collected data for each hospital ward on the same day.

### 4.2. Data Collection

Each country assigned a national coordinator who provided general oversight to complete the survey and ensure appropriate good-quality data collection. Each participating hospital assigned a hospital coordinator supported by an investigator team composed of hospital infection control staff, clinical pharmacists, and medical doctors. WHO trained all staff engaged with data collection on the use of the study protocol, data collection procedures, data entry, and medical ethics. Hospital teams collected information on hospital affiliation (public, private, and academia), hospital size, admission specialty, age, and gender. For patients receiving an antimicrobial agent, the teams collected information from the medical records on the type, dose, indication for prescription, and site of infection. We classified antimicrobial indications as: (i) treatment of community-acquired infection (CAI) if symptoms were present on admission; (ii) treatment of hospital-acquired infection (HAI) if symptoms started 48 h after admission; (iii) medical prophylaxis (MP), which includes prevention of bacterial infections in patients with late-stage cirrhosis, upper gastrointestinal bleeding, and acute necrotizing pancreatitis and prevention of opportunistic infections in immunocompromised patients (e.g., HIV/AIDS patients); (iv) surgical prophylaxis (SP); (v) other; and (vi) unknown.

We collected five prescribing quality indicators including (i) availability and compliance with hospital treatment guidelines (Alignment of the choice of antibiotic with the clinical guidelines at the facility (national or local guideline), (ii) documentation of the reason for antimicrobial use, (iii) stop/review date of antimicrobials, (iv) route of administration (parental, oral, inhalation), and (v) targeted (antimicrobial prescribed in response to microbiology results) or empirical treatment.

### 4.3. Data Entry

We developed an electronic application to facilitate automated data entry and reporting and reduce errors by including built-in complex validation and skip logic rules. The hospital coordinators monitored the progress in data collection and validated and revised the quality of data entered before final submission to the national team.

### 4.4. Data Analysis

We analyzed the data using STATA version 16 [38]. We estimated the prevalence of antimicrobial use as the proportion of patients receiving at least one antimicrobial agent on the day of the survey. The denominator was the number of all patients hospitalized on the day of the survey before or at 8:00 am. We classified the types of antimicrobials according to the internationally recognized WHO anatomical therapeutic chemical classification system (ATC) for the classification of drugs at the level of the chemical group [39]. In Table 1, we analyzed and presented only the most common antimicrobial groups prescribed, which constituted 90% of the total antimicrobial prescriptions.

We classified the antibacterials prescribed according to the WHO Access, Watch, Reserve (AWaRe) classification, where the Access group contains narrow-spectrum antibacterials recommended as first and second choice for most common clinical infection syndromes. The Watch group contains broader spectrum antibacterial classes. The Reserve group consists of last-resort antibacterials for targeted use in multidrug-resistant infections [40]. 

We used box-and-whisker plots to present the proportion of patients prescribed antimicrobials per country, where the horizontal line inside the box showed the median proportion of patients prescribed antimicrobials and the upper and lower end of each box provide the 75th and 25th interquartile ranges. The area between the different parts of the box indicated the degree of dispersion and skewness of data. The ends of the whiskers represented the minimum and maximum percentage of patients that were prescribed antimicrobials

## 5. Conclusions

The high prevalence of antimicrobial prescription among hospitalized patients with use of broad-spectrum antibiotics with no clear indications reflects the infancy of AMS programs in several EM countries. None of the countries achieved the target of 60% antimicrobial use from Access group, with a high prescription of Watch antimicrobials, and there is low compliance with several quality indicators of antimicrobial prescribing. The situation calls for ministries of health leadership to enhance the design and implementation of national and facility stewardship programs and provide the necessary human and financial resources for country-wide implementation. Countries must use their data on the prevalence of antimicrobial use together with other national consumption and resistance data to design and implement tailored antimicrobial stewardship programs with specific quality improvement targets. They should adopt the AWaRe classification as a stewardship tool into essential medicine lists and national treatment guidelines to increase the utilization of narrow-spectrum antibiotics and preserve the precious Reserve group. While the development and implementation of national standardized treatment guidelines for infectious diseases seem to be an overall priority for countries, additional AMS strategies must also be implemented, e.g., education and training of physicians on appropriate prescribing practices is crucial. Changing practices and prescribing patterns of health care providers will take time, but investment is critical as a long-term solution. Regular point prevalence surveys should monitor the impact of antimicrobial stewardship interventions. Strong infection prevention and control programs must prevent the spread of resistance, and the surveillance of antimicrobial resistance must be sustained.

## Figures and Tables

**Table 1:** Distribution of prescribed antimicrobial groups, seven countries in the Eastern Mediterranean Region, 2019.

Antimicrobial Group/Subgroup	Overall		Tunisia		UAE		Lebanon		Jordan		Sudan		Iraq		Pakistan	
	n	%	n	%	n	%	n	%	n	%	n	%	n	%	n	%
**Third-generation cephalosporins**	4516	26.7	652	20.7	502	17.2	340	16.7	840	28.6	291	31.7	296	38.4	1595	38.5
**Beta-lactam antibacterials, Penicillins (J01C)**	3055	18.1	874	27.7	597	20.4	376	18.5	358	12.2	78	8.5	45	5.8	727	17.5
**Imidazole derivatives**	1661	9.8	264	8.4	158	5.4	142	7	286	9.7	174	18.9	153	19.8	484	11.7
**Carbapenems**	1384	8.2	212	6.7	225	7.7	252	12.4	287	9.8	37	4	80	10.4	291	7
**Fluoroquinolones**	1241	7.4	383	12.2	163	5.6	196	9.6	123	4.2	75	8.2	48	6.2	253	6.1
**Aminoglycosides**	941	5.6	219	6.9	99	3.4	57	2.8	185	6.3	58	6.3	44	5.7	279	6.7
**Glycopeptides**	920	5.4	127	4	128	4.4	173	8.5	223	7.6	67	7.3	33	4.3	169	4.1
**Second-generation cephalosporins**	602	3.6	17	0.5	147	5	81	4	293	10	57	6.2	-	-	7	0.2
**Macrolide, Lincosamides, Streptogramins**	546	3.2	49	1.5	182	6.2	68	3.3	114	3.9	15	1.6	27	3.5	91	2.2
**First-generation cephalosporins**	525	3.1	65	2.1	189	6.5	110	5.4	136	4.6	-	-	1	0.1	24	0.6
**Total**	15391	91.2	2862	90.8	2390	81.7	1795	88.2	2845	96.9	852	92.8	727	94.3	3920	94.6

**Table 2:** Quality indicators of antimicrobial prescribing, seven countries in the Eastern Mediterranean Region, 2019.

Country	Treatment Guidelines					Indication Documented			Stop/Review Order Documented			Injectable Administration			Targeted Treatment		
		Guidelines Available		Compliant to Guidelines *													
	N	n	%	n	%	N	n	%	N	n	%	N	n	%	N	n	%
Overall	16,697	6841	41	5070	74	16,409	9987	61	16,117	4843	30	16,763	14,480	86	9179	1919	21
UAE	2930	2406	82	2067	86	2930	2557	87	2930	2121	72	2927	2172	74	1867	602	32
Lebanon	1898	1223	64	1021	84%	1866	1378	74	2050	211	10	1990	1752	88	1741	393	23
Tunisia	3049	439	14	375	85	3061	1886	62	3109	617	20	3114	2639	85	2452	524	21
Jordan	2870	968	34	386	40	2734	1321	48	2842	1504	53	2909	2613	90	1105	184	17
Sudan	1073	221	21	161	73	941	565	60	1035	255	25	901	827	92	842	142	17
Pakistan	4151	1534	37	1015	66	4151	2191	53	4151	135	3	4151	3818	92	1172	74	6
Iraq	726	50	7	45	90	726	89	12	-	-	-	771	659	85	-	-	-

* The number of antimicrobials prescriptions for which guidelines were available was used as the denominator to calculate percentage.

## Data Availability

Available on reasonable request from the corresponding authors.
